# Patient-Reported Outcome questionnaires for hip arthroscopy: a systematic review of the psychometric evidence

**DOI:** 10.1186/1471-2474-12-117

**Published:** 2011-05-27

**Authors:** Marsha Tijssen, Robert van Cingel, Nicky van Melick, Enrico de Visser

**Affiliations:** 1 Sport Medisch Centrum Papendal, Papendallaan 7, 6816 VD, Arnhem, The Netherlands; 2 HAN University of Applied Sciences, Institute Health Studies, Kapittelweg 33, 6525 EN Nijmegen, The Netherlands; 3 Department of orthopedics, Rijnstate Hospital, Wagnerlaan 55, 6816 AD Arnhem, The Netherlands

## Abstract

**Background:**

Hip arthroscopies are often used in the treatment of intra-articular hip injuries. Patient-reported outcomes (PRO) are an important parameter in evaluating treatment. It is unclear which PRO questionnaires are specifically available for hip arthroscopy patients. The aim of this systematic review was to investigate which PRO questionnaires are valid and reliable in the evaluation of patients undergoing hip arthroscopy.

**Methods:**

A search was conducted in Pubmed, Medline, CINAHL, the Cochrane Library, Pedro, EMBASE and Web of Science from 1931 to October 2010. Studies assessing the quality of PRO questionnaires in the evaluation of patients undergoing hip arthroscopy were included. The quality of the questionnaires was evaluated by the psychometric properties of the outcome measures. The quality of the articles investigating the questionnaires was assessed by the COSMIN list.

**Results:**

Five articles identified three questionnaires; the Modified Harris Hip Score (MHHS), the Nonarthritic Hip Score (NAHS) and the Hip Outcome Score (HOS). The NAHS scored best on the content validity, whereas the HOS scored best on agreement, internal consistency, reliability and responsiveness. The quality of the articles describing the HOS scored highest. The NAHS is the best quality questionnaire. The articles describing the HOS are the best quality articles.

**Conclusions:**

This systematic review shows that there is no conclusive evidence for the use of a single patient-reported outcome questionnaire in the evaluation of patients undergoing hip arthroscopy. Based on available psychometric evidence we recommend using a combination of the NAHS and the HOS for patients undergoing hip arthroscopy.

## Background

Hip arthroscopy is a relatively new procedure in the management of hip disorders [1,2]. It has first been described by Burman [3] in 1931, but has not evolved into general use since approximately the last two decades [4]. The indications for hip arthroscopy are numerous and include, symptomatic labral tears, femoroacetabular impingement (FAI), loose bodies, synovitis, chondral defects and degenerative conditions of the hip [4,5]. This broad range of indications also implies a broad range of patients [6,7]. Arthroscopies are performed on adolescents and professional athletes, but also on older populations (<55 years) [2,7-9]. Exact numbers on incidence and prevalence of these surgical interventions are unknown.

The number of hip arthroscopies is rising because of improvements in surgical technique and a better understanding of the pathology associated with the hip joint [10]. Therefore, the need for outcome related research increases [10]. One important parameter in outcome-related research in all areas of medicine is the patient's perspective [11]. As Patrick et al. [11] described patient-reported outcomes (PROs) should serve as a golden standard in the assessment of musculoskeletal conditions where the patients perspective and health-related quality of life are of main interest.

A number of PRO questionnaires have been developed for individuals with hip pathology, especially osteoarthritis [12-14]. The small amount of outcome related research available for hip arthroscopy uses many of these different questionnaires, but it is unclear if these are valid and reliable in the assessment of patients undergoing hip arthroscopy [12]. In order to recommend or discard these PRO questionnaires analysis of their content and psychometric properties is necessary. Thus far, two systematic reviews in this area have been performed [13,14]. Schenker et al. [13] concluded that the Hip Outcome Score (HOS) was the most reliable and valid measure of self-reported physical function for individuals undergoing hip arthroscopy. It is unclear which methods were used to achieve this conclusion and which questionnaires and psychometric evidence were compared. Furthermore, the review only provides evidence for the HOS in pre-operative use [13]. The second study by Thorborg et al. [14] reviewed all questionnaires assessing hip and groin disability on validity, reliability and responsiveness and concluded that the HOS should be recommended for evaluating patients undergoing hip arthroscopy. This conclusion was based on the number of psychometric properties known for the particular questionnaires involved in the study [14]. More psychometric properties meant a better quality questionnaire. However, the quality of studies investigating the psychometric evidence was not a subject of research, which could possibly lead to bias [15].

The aim of this systematic review was to investigate which PRO questionnaires are valid and reliable in the evaluation of patients undergoing hip arthroscopy.

## Methods

A systematic review was performed 1) to identify all PRO questionnaires used in the evaluation of patients undergoing hip arthroscopy 2) to evaluate the quality of these questionnaires based on their psychometric evidence 3) to determine the methodological quality of the studies into the psychometric evidence of these questionnaires.

### Definitions

A health-related PRO questionnaire is a measurement of any aspect of a patient's health status that is directly assessed by the patient, thus without interpretation of the patient's responses by a physician or anyone else [15].

Psychometric properties are part of psychometrics, which is the discipline concerned with the construction and validation of measurement instruments, such as questionnaires and tests [16]. The psychometric properties used in this study are defined by Terwee et al. [17] and consist of: content validity, internal consistency, criterion validity, construct validity, agreement, reliability, responsiveness, floor and ceiling effects and interpretability.

### Search Strategy

A computerized literature search was performed using Pubmed, Medline, CINAHL (via EBSCO), the Cochrane Library, Pedro, EMBASE (via OVID) and Web of Science to identify relevant articles published between January 1931 and 1 October 2010. The search was conducted by two reviewers (NM and MT). The following terms were used:

Hip AND arthroscopy

Hip AND arthroscopy AND questionnaires OR outcome assessment OR self assessment OR outcome

Hip AND rehabilitation OR treatment AND questionnaires OR outcome assessment OR self assessment OR outcome

Terms were searched as key words or 'free-text' terms in all databases except for Pubmed in which they were searched as MESH terms. The reference lists of the retrieved articles were searched for more relevant studies. The search was completed with a separate search for the identified questionnaires as well as for authors of these questionnaires.

### Study Selection

**Figure 1:**
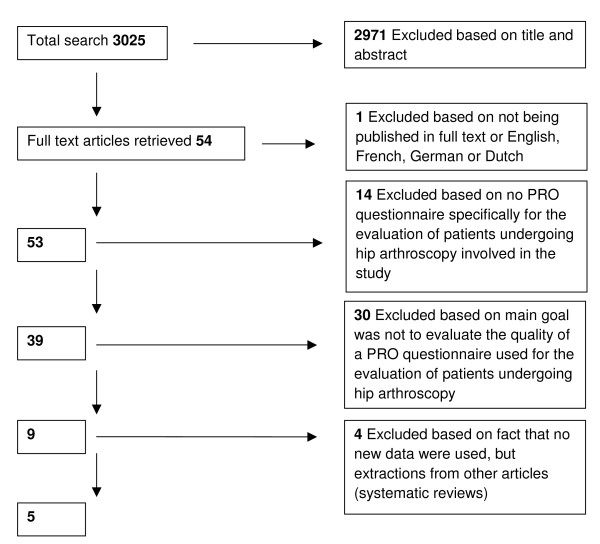
Selection of publications with exclusion criteria

The two reviewers (NM and MT) independently assessed all collected publications on title and abstract for possible inclusion in the study. All selected publications were retrieved in full and in- and exclusion criteria were applied by the two reviewers. Inclusion criteria are presented in Table 1. Disagreements between reviewers were resolved by consensus. If consensus was not reached the final decision was made by a third reviewer (RC). The reviewers were not blinded to authors, date of publication and journal of publication. An overview of the selection procedure and exclusion criteria is presented in Figure 1. Exclusion criteria directly assessable from title and abstract were evaluated first and the criteria that needed thorough examination of the article were evaluated secondly.

**Table 1:** Inclusion criteria

Inclusion criteria
1. Article was published in English, French, German or Dutch and available as full text article.

2. The study included a PRO questionnaire specifically used for the evaluation of patients following hip arthroscopy

3. The main goal of the study was to evaluate the quality of a PRO questionnaire used for the evaluation of patients undergoing hip arthroscopy

4. The study used new data instead of data extracted from other research (for example systematic reviews)

### Quality Assessments

Two assessment procedures were used to assess the quality of the identified questionnaires and the methodological quality of the articles describing the questionnaires.

Terwee et al. [17] developed quality criteria for good psychometric properties in order to evaluate and compare the quality of PRO questionnaires. The list contains the following items: content validity, internal consistency, criterion validity, construct validity, reproducibility (agreement/reliability), responsiveness, floor and ceiling effects and interpretability [17]. The items are rated as positive (+), intermediate (?), negative -, or no information available (). The exact definitions of the psychometric properties and scoring criteria can be found in Additional file 1.

No overall score is calculated, but a conclusion is drawn based on the information of the properties combined with the aim of the questionnaire [17]. This criteria list was used in previous systematic reviews [14,18]. The reviewers (MT and NM) rated the articles independently in order to avoid systematic errors.

**Figure 2:**
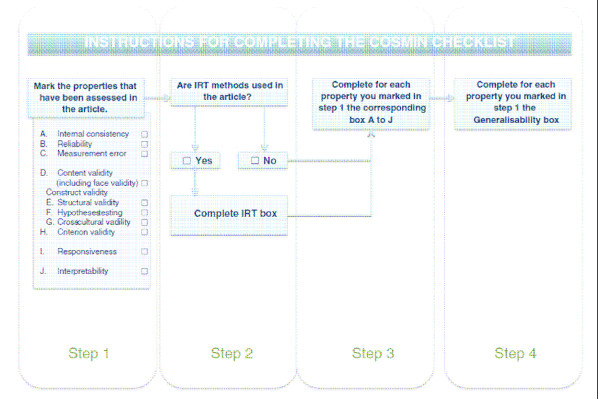
**COSMIN checklist**. The 4-step procedure to complete the COSMIN checklist for evaluating the quality of studies investigating the psychometric properties of health-related PRO questionnaires [15].

The methodological quality of the studies into the psychometric evidence of these questionnaires was determined by the Consensus-based Standards for the selection of health Measurement Instruments list (COSMIN) [15]. This list has recently been developed and published by Mokkink et al. [15]. The COSMIN list is based on an international Delphi study in which 57 experts participated and has proven to have a good inter-rater agreement and reliability [15,19]. It contains four steps and 12 boxes (Figure 2). Ten boxes can be used to assess whether a study meets the standard for good methodological quality (Boxes A to J) [15]. In addition, two boxes are included containing general requirements for articles in which Item Response Theory (IRT) methods and general requirements for the generalisability of the results are applied [15]. Only the boxes corresponding with the properties assessed in the study should be evaluated. The generalisability box should be evaluated for each psychometric property as one article may use different study populations for different properties [15]. Each item is rated as excellent (+++), good (++), fair (+) or poor (0). The overall score per box is determined by the item with the lowest score. The reviewers (NM and MT) conducted the review process in the same manner as with the quality assessment of the questionnaires.

## Results

The total search identified 3025 articles. A total of 2971 articles were excluded based on title and abstract which left 54 articles that were read in full text. Of these 54 articles 49 were excluded based on the remaining exclusion criteria which left five articles to be included in the study with a total of 830 subjects. None of the articles used the same group of subjects for their data collection. An overview of the descriptive data of the articles is shown in Table 2. The search identified three different questionnaires regarding the management of patients following hip arthroscopy: the Modified Harris Hip Score (MHHS), the Nonarthritic Hip Score (NAHS) and the Hip Outcome Score (HOS) (Table 3).

**Table 2:** Descriptive data of the 5 selected articles

Authors (Year)	Questionnaire	Study Population	Time of administration	Target Population
Chirstensen et al. (2003) [21]	NAHS	Hip pain >6 months, no abnormalities RX N = 48/17 19♂, 29♀/6♂, 11♀ 33y (range 16-45)/32y	Clinical visit	Young patients with hip pain pre- and postoperative

Martin et al. (2006) [22]	HOS	Labral tear N = 507 (263 operation) 232♂, 273♀ 38y (SD 13y, range 13-66)	Pre-operative	Patients with labral tears (conservative + operative)

Martin et al. (2007) [23]	HOS	Hip arthroscopy N = 107 51♂, 56♀ 42y (SD 14, median 44.2, range 14-79)	Post-operative follow-up 3.1y (SD 0.49, range 2-4.6)	Hip arthroscopy patients >2 years

Martin et al. (2008) [24]	HOS	Hip arthroscopy N = 126 59♂, 67♀ 41y (SD 16, range 13-80)	Pre-operative. Post-operative 7 months	Hip arthroscopy patients

Potter et al. (2005) [20]	MHHS	Hip arthroscopy labral tears N = 33 14♂, 19♀ 34.6y (range 21-56y)	Post-operative mean follow-up 25.7 months (range 13-55 months)	Hip arthroscopy patients - labral tears

**MHHS = Modified Harris Hip Score. NAHS = Nonarthritic Hip Score. HOS = Hip Outcome Score. N = number of subjects involved in the study./= different study population. Y = years. SD = standard deviation. RX = radiographs. Target Population = target population as used in articles**.

**Table 3:** Descriptive data of questionnaires

Question-naire	Aim	Measurement Dimensions	Target Population	Rating Scales	Nr. Questions
MHHS	Evaluative Measure pre/post-operative hip pain and function	Pain, function, functional activities	Hip arthroscopy patients	2	8

NAHS	Evaluative Measure pre/post-operative hip pain and function	Functional activities, pain, symptoms, sports	20 - 40 year old patients with hip pain and without radiographic diagnosis	1	24

HOS	Evaluative Measure outcome treatment intervention	Functional activities, sports	Subjects with acetabular labral tears with function of wide range of ability	2	26

**MHHS = Modified Harris Hip Score. NAHS = Nonarthritic Hip Score. HOS = Hip Outcome Score. Measurement dimensions = as stated in questionnaire. Target Population = as described by authors designing questionnaires**.

Any disagreement between the two reviewers (NM and MT) was resolved by consensus.

### **Quality of questionnaires and articles**

The psychometric properties per questionnaire are shown in Table 4. The scores of the individual articles as assessed by the reviewers (NM and MT) can be found in Additional file 2: Quality of the questionnaires based on psychometric properties rated by article.

**Table 4:** Quality of the questionnaires based on psychometric properties

Question-naire	Content validity	Internal consistency	Criterion validity	Construct validity	Reproducibility (Agreement)	Reproducibility (Reliability)	Respon-siveness	Floor and ceiling effects	Interpretability
MHHS	()	()	()	+	()	()	()	()	?

NAHS	+	?	()	?	()	?	()	()	()

HOS	-	+	()	+	+	+	+	?	?

**+ = positive rating, ? = intermediate rating, - = negative rating, () = no information available. For exact information on content of psychometric properties see Terwee et al. **[17].

The MHHS scored high on construct validity because it correlated well with the domains bodily pain and physical functions of the Short Form-36 (SF-36) [20]. Some information on interpretability is known, however this information was not comprehensive and therefore this property scored an intermediate rating [20]. The NAHS scored high on content validity, but intermediate on internal consistency, construct validity and reproducibility. The internal consistency was checked with a factor analysis but this was performed with too little subjects [21]. A Pearson Correlation Coefficient was used to check for reliability instead of an ICC or Kappa [21]. The correlation between the NAHS and the SF-12 on the physical and emotional domains was good, but not in compliance with the a priori formulated hypothesis and thus let to an intermediate rating for construct validity [21]. The HOS scored good on internal consistency, construct validity, agreement, reliability and responsiveness. However, because no target population was used, the content validity was rated negative [22-24]. The construct validity was checked with a SF-36 and a rating scale for level of function and surgical outcome. Only the correlation with the SF-36 was used to establish construct validity, which was good [22,23]. Remarkably, the construct validity of all questionnaires was checked with either a SF-36 or SF-12 [20-23]. Furthermore, for none of the questionnaires definite information was available for criterion validity, floor and ceiling effects and interpretability.

The quality of the articles investigating the psychometric evidence of the PRO questionnaires were rated by the COSMIN checklist and presented in Table 5. The MHHS was investigated by Potter et al. [20]. The generalisability and construct validity, measured by hypothesis testing, was good. The NAHS was investigated by Christensen et al. [21]. The test-retest reliability was rated poor because the time interval differed from 1 to 16 days and no information was available on possible changes in the patients complaints. All other parameters were rated as fair because of some information lacking per parameter. The three articles investigating the HOS were all published by Martin et al. [22-24]. The first study was the only one that used IRT in the development of the questionnaire [22]. The hypothesis testing and structural validity were rated fair because patients who could not answer enough questions of the HOS were excluded for analysis, leading to possible bias. Martin et al. [23] tested the construct validity of the HOS again for hip arthroscopy patients were it was developed for evaluating the treatment of acetabular labral tears, but the overall generalisability of this study was less than in the previous investigation. The reliability and responsiveness were investigated with a total of 126 subjects [24]. These were divided into 18 stable versus 108 changed subjects. This led to a fair to poor score for the quality of these measurement properties and generalisability. Overall the generalisability box scored better than the quality of the assessment of the properties per article.

**Table 5:** Scores of articles rated by COSMIN checklist

Authors (year)	Measurement properties assessed	IRT used	Score IRT	A*	B*	C*	D*	E*	F*	G*	I*	J*	Generalisability per box
Chirstensen et al. (2003) [21]	Internal consistency Reliability Content validity Hypotheses testing	No		+	0		+		+				++ A ++ B ++ D ++ F

Martin et al. (2006) [22]	Internal consistency Hypothesis testing Structural validity	Yes	++	++				+	+				+++ IRT +++ A +++ E +++ F

Martin et al. (2007) [23]	Hypothesis testing Structural validity Interpretability	No						++	+			++	++ E ++ F ++ J

Martin et al. (2008) [24]	Reliability Responsivenss Interpretability	No			+						+	+	0 B ++ I ++ J

Potter et al. (2005) [20]	Construct validity	No							+++				+++ F

+++ = excellent. ++ = good. + = fair. 0 = poor. Empty boxes = not applicable. IRT = Item Response Theory. * = A = internal consistency. B = reliability. C = measurement error. D = content validity. E = structural validity. F = hypothesis testing. G = cross-cultural validity. H = criterion validity. I = responsiveness. J = interpretability.

## Discussion

This systematic review included five articles on hip arthroscopy using three different questionnaires (NAHS, HOS and MHHS). The MHHS is a modification of the Harris Hip Score which is an observer-administrated score [25]. Potter et al. [20] used it as a self-administrated score, deleting the two observer-administrated items. Therefore the MHHS was included in this study. In previous studies more questionnaires were used but these were often developed for osteoarthritis [12,13,26,27]. Furthermore, none of these studies explicitly investigated the quality of the questionnaires used for the evaluation of hip arthroscopy patients [12,13,26,27]. The quality of the questionnaires was assessed by the criteria list of Terwee et al. [17]. The methodological quality of the studies into the questionnaires was assessed by the COSMIN list [15]. Based on the quality criteria proposed by Terwee et al. [17] none of the three identified questionnaires had a high quality. Not all measurement properties are equally important for the quality of a questionnaire [17]. Terwee et al. [17] considered the content validity to be one of the most important measurement properties and stated that only if this is adequate, one should consider using a questionnaire. Based on this parameter the NAHS would be the best quality questionnaire. However, they also showed that the aim of the questionnaire demands different qualities of a questionnaire and thus measurement properties [17]. As all three included questionnaires were evaluative a high level of agreement was important. In that perspective the HOS scored the best.

The overall quality of the articles investigating the measurement properties as rated by the COSMIN list was fair to good. Remarkably, in most cases the generalisability per box was better than the quality of the assessed properties per article. Only one article scored excellent on hypothesis testing of the correlation between the MHHS and SF-36 [20]. Furthermore, two articles by Martin et al. [22,23] examining the validity of the HOS had one or more scores that were rated good. When adding all scores the article by Martin et al. [22] had the highest quality.

The NAHS has been developed for a young population with orthopedic, non arthritic hip pain and not specifically for patients undergoing hip arthroscopy, like the HOS [12]. Therefore, the NAHS may be a more generalisable questionnaire, but less specific for hip arthroscopy patients. Studies investigating the HOS excluded subjects that could not answer a certain amount of questions, which could lead to bias [22,23]. Furthermore, the HOS has a sports subscale which may fit an athletic population but may not be appropriate for individuals with slight degenerative conditions undergoing hip arthroscopy [12]. These two disadvantages may compromise the reliability and validity of the HOS. Evidence for the support of the NAHS as well as the HOS can be found in other systematic reviews [12-14]. Baldwin et al. [12] performed a review concerning the outcomes of hip arthroscopy for the treatment of FAI and concluded that the NAHS was the most suitable scale for evaluating FAI. However, the quality assessment in this article was performed based on the authors experience and preference. The HOS was found the best in the assessment performed by Schenker et al.[13] and Thorborg et al. [14]. Yet, Schenker et al. [13] did not define the search strategy nor the identified questionnaires and the methods on which they based their quality assessment. Thorborg et al. [14] used only the amount of measurement properties per questionnaire and not the quality of the articles investigating it. Further, they used the criteria stated by Terwee et al. [17] for the evaluation of measurement properties of PRO questionnaires in hip arthroscopy patients, but found different results due to interpretation differences. This was foreseen by Terwee et al. [17] who stated that at least the criteria list would separate poor from good quality questionnaires. Based on this separation our review stated the MHHS to be of moderate quality and the NAHS and HOS to be of better quality. Thorborg et al. [14] stated the HOS to be of good quality.

The COSMIN list we used in this review was recently developed. At present no other checklists for the assessment of articles on the methodological quality of questionnaires are available [15,16,19]. There is also no list that scores both the quality of the questionnaires and the quality of the studies investigating the questionnaires [15]. Therefore, a combination of the list by Terwee et al. [17] and the COSMIN list has been recommended in assessing the quality of questionnaires [15]. Using these two lists we concluded that the NAHS is the best quality questionnaire, but the quality of the articles describing the HOS is higher. The quality of a systematic review depends on the quality of the studies included. A limitation of this study is the small number of questionnaires as well as the small number of studies that could be included. More rigorous studies to determine which score is most valid and reliable are necessary to provide a conclusive recommendation.

## Conclusions

This systematic review shows that there is no conclusive evidence for the use of a single patient-reported outcome questionnaire in the evaluation of patients undergoing hip arthroscopy. A limitation of this study is the small number of studies that could be included. Based on available psychometric evidence we recommend using a combination of the NAHS and the HOS for patients undergoing hip arthroscopy. In order to provide a conclusive recommendation more studies on the validity and reliability of these questionnaires are warranted.

## Competing interests

The authors declare that they have no competing interests.

## Authors' contributions

MT participated in the design of the study, carried out the literature search, selection and evaluation of articles and writing of the review. RC participated in the design of the study and revising the manuscript. NM carried out the literature search, selection and evaluation of articles. EV participated in the design of the study and revising the manuscript. All authors read and approved the final manuscript.

## Pre-publication history

The pre-publication history for this paper can be accessed here:

http://www.biomedcentral.com/1471-2474/12/117/prepub

## Supplementary Material

Additional file 1 **Definitions and scoring criteria of the psychometric properties**. Definitions and scoring criteria of the psychometric properties developed by Terwee et al. Note: Important for other authors in order to get a clear image of the research performed. Not important enough to be placed in manuscript. Click here for file

Additional file 2 **Quality of the questionnaires based on psychometric properties rated by article**. Quality of the questionnaires based on psychometric properties and displayed by article. Note: Important for other authors in order to get a clear image of the research performed. Not important enough to be placed in manuscript. Click here for file
