# Supplementary material for: Patient-Reported Outcome questionnaires for hip arthroscopy: a systematic review of the psychometric evidence
Source: BMC Musculoskelet Disord. 2011 May 27;12:117. doi: 10.1186/1471-2474-12-117 (PMC3129322; doi:10.1186/1471-2474-12-117)
Supplement: Additional file 1 — Definitions and scoring criteria of the psychometric properties. Definitions and scoring criteria of the psychometric properties developed by Terwee et al. Note: Important for other authors in order to get a clear image of the research performed. Not important enough to be placed in manuscript. [file 1471-2474-12-117-S1.DOC]

**Additional file 1 - Definitions and scoring criteria of the psychometric properties**

For exact information on content of psychometric properties see Terwee et al. [17].
