# Supplementary material for: Patient-Reported Outcome questionnaires for hip arthroscopy: a systematic review of the psychometric evidence
Source: BMC Musculoskelet Disord. 2011 May 27;12:117. doi: 10.1186/1471-2474-12-117 (PMC3129322; doi:10.1186/1471-2474-12-117)
Supplement: Additional file 2 — Quality of the questionnaires based on psychometric properties rated by article. Quality of the questionnaires based on psychometric properties and displayed by article. Note: Important for other authors in order to get a clear image of the research performed. Not important enough to be placed in manuscript. [file 1471-2474-12-117-S2.DOC]

**Additional file 2 - Quality of the questionnaires based on psychometric properties rated by article**

| **Authors** | **Content validity** | **Internal consistency** | **Criterion validity** | **Construct validity** | **Reproducibility (Agreement)** | **Reproducibility (Reliability)** | **Responsi-veness** | **Floor and ceiling effects** | **Interpretability** |
| --- | --- | --- | --- | --- | --- | --- | --- | --- | --- |
| Chirstensen et al. 2003  [22] | + | ? | () | ? | () | ? | () | () | () |
| Martin et al. 2006 [23] | - | + | () | + | () | () | () | () | () |
| Martin et al. 2007 [24] | () | () | () | + | () | () | () | () | () |
| Martin et al. 2008 [25] | () | () | () | () | + | + | + | ? | ? |
| Potter et al. 2005 [21] | () | () | () | + | () | () | () | () | ? |

+ = positive rating, ? = intermediate rating, - = negative rating, () = no information available. For exact information on content of psychometric properties see Terwee et al. [17].
